# A decision support tool to enhance agricultural growth in the Mékrou river basin (West Africa)

**DOI:** 10.1016/j.compag.2018.09.037

**Published:** 2018-11

**Authors:** Angel Udias, Marco Pastori, Céline Dondeynaz, Cesar Carmona Moreno, Abdou Ali, Luigi Cattaneo, Javier Cano

**Affiliations:** a European Commission–Joint Research Centre, via E. Fermi 2749, 21027 Ispra, VA, Italy; b AGRHYMET Regional Centre, Niamey, Niger; c Universidad Rey Juan Carlos, Madrid, Spain; d University of Auckland, Auckland, New Zealand

**Keywords:** Multiobjective optimization, Food security, Best management practices, African agricultural growth, WEFE nexus

## Abstract

We describe in this paper the implementation of E-Water, an open software Decision Support System (DSS), designed to help local managers assess the Water Energy Food Environment (WEFE) nexus. E-Water aims at providing optimal management solutions to enhance food crop production at river basin level. The DSS was applied in the transboundary Mékrou river basin, shared among Benin, Burkina Faso and Niger. The primary sector for local economy in the region is agriculture, contributing significantly to income generation and job creation. Fostering the productivity of regional agricultural requires the intensification of farming practices, promoting additional inputs (mainly nutrient fertilizers and water irrigation) but, also, a more efficient allocation of cropland.

In order to cope with the heterogeneity of data, and the analyses and issues required by the WEFE nexus approach, our DSS integrates the following modules: (1) the EPIC biophysical agricultural model; (2) a simplified regression metamodel, linking crop production with external inputs; (3) a linear programming and a multiobjective genetic algorithm optimization routines for finding efficient agricultural strategies; and (4) a user-friendly interface for input/output analysis and visualization.

To test the main features of the DSS, we apply it to various real and hypothetical scenarios in the Mékrou river basin. The results obtained show how food unavailability due to insufficient local production could be reduced by, approximately, one third by enhancing the application and optimal distribution of fertilizers and irrigation. That would also affect the total income of the farming sector, eventually doubling it in the best case scenario. Furthermore, the combination of optimal agricultural strategies and modified optimal cropland allocation across the basin would bring additional moderate increases in food self-sufficiency, and more substantial gains in the total agricultural income.

The proposed software framework proves to be effective, enabling decision makers to identify efficient and site-specific agronomic management strategies for nutrients and water. Such practices would augment crop productivity, which, in turn, would allow to cope with increasing future food demands, and find a balanced use of natural resources, also taking other economic sectors—like livestock, urban or energy—into account.

## Highlights

• An effective DSS integrating several models and methods has been developed.
• The E-water tool enable the identification of site-specific agronomic practices for nutrients and water management.
• Identified optimal solutions take into account food demand thus coping with food security issue.
• The main features of the DSS are tested by applying it to various scenarios in the Mékrou river basin.

## 1 Introduction

The World Bank reports that, although the proportion of African population living under the extreme poverty line has dropped off in the last years (Beegle et al., 2016), food scarcity is still a major challenge, especially in Sub-Saharan countries, and, in particular, in rural areas where the main source of livelihood is agriculture (Markantonis et al., 2017a). This is reflected in the predominant contribution of agriculture and agro-related activities to income generation (around 30% of GDP globally in the region) and job creation (78% in Burkina Faso, 45% in Benin and 57% in Niger) (World Bank, Development Research Group, 2017).

Within this context, agriculture is characterized by a meager productivity with poor levels of intensification. Therefore, developing more efficient agricultural techniques is vital to enhance prosperity and alleviate poverty, especially in rural areas. Indeed, it is well known that poverty reduction and agricultural productivity are strongly correlated (Thirtle and Piesse, 2007). Other studies show, in turn, that improvements in agricultural performance have paid in important benefits to rural population, by increasing farmers’ incomes, job opportunities and wages, being the latter particularly critical for impoverished households (Dorward, 2003, Poulton and Dorward, 2003).

To address all these issues, a cooperation project involving the Joint Research Centre of the European Commission and the Global Water Partnership West Africa was set. Food insecurity was identified as a major concern for the whole region, especially in the northern area, belonging to Niger. Interestingly, those regions with a more pronounced food insecurity problem, were also those with lower agricultural productions reported, due to modest yields and/or lack of fertile land available (Markantonis et al., 2017b, Markantonis et al., 2018).

Several agronomic studies suggest that the management of poor soils is the main limiting factor for agricultural production (Van Keulen and Breman, 1990). However, due to its scarce and extremely variable nature, rainfall has also a major influence in rainfed agriculture. Therefore, climate change and its effects should also be taken into account when considering future developments, since the use of additional water, combined with improved fertilization management, could entail important benefits to local crop production. Other factors that could potentially affect agricultural growth are economic (accessibility to fertilizers), droughts, floods and missing land. The latter is most likely due to the inadequate management of soil fertility—which may turn land unfertile, being, eventually, abandoned by local farmers—and, to a lesser extent, to pressures from the livestock sector, which demands land for animal pasture. Summarizing, farmers’ ability to yield, at a local scale, crop products needed to elaborate food, feed their animals and, more generally, increase their income and wellbeing is greatly limited by the aforementioned determinants.

On the other hand, in the Mékrou river basin, yields are mainly constrained by the low availability of nutrients, being water less influential, since the dominant agriculture is rainfed and produced during the rainy season. Nevertheless, the prevailing pattern observed in the region actually exhibits a simultaneous limitation of nutrients and water for crop yields.

Within this framework, policies stimulating an escalation of agriculture—in terms of a more intense and efficient use of nutrients and water—are clearly required. However, it is not evident how to minimize their implementation costs: therefore the need to advise local managers and stakeholders on how to optimize their investment decisions for each combination of crops and regions.

In this regard, optimization techniques have previously been applied to the management of agriculture farms under different perspectives (Plà et al., 2014). For instance, Hassan et al. (2005) reported that linear programing (LP) allows to devise equilibrium solutions between arable crop based farms and livestock. In another work Ohajianya and Oguoma (2009) analyzed resource allocation patterns for 120 food crops farms in Imo State, Nigeria, using LP to optimize resources. Alternatively, Ibrahim et al. (2009) relied on LP to determine optimal farm plans to evaluate the food security status of farming households. In turn, Majeke and Majeke, 2010, Harasimowicz et al., 2017 dealt with the farm resource allocation problem, observing that better results were obtained when using an LP model instead of the traditional planning method. In Andreea and Adrian (2012), an LP method was proposed to determine the optimal structure of crops. A multiobjective approach, considering increasing productivity and the environmental impact, has frequently been applied to farms management (Pastori et al., 2017, Plà et al., 2014, Galán-Martín et al., 2017, Groot et al., 2012). However, most mathematical programming methods in the agronomic field have limited applicability, being in practice unavailable to stakeholders involved in the development of generic agricultural, growth, or environmental policies. Furthermore, they usually focus on a single type of analysis, as e.g. irrigation, fertilization or land reassignment.

Aimed at overcoming such limitations, DSSs are computer software programs that use models, data and other related information to make site-specific recommendations with various purposes. Recent examples in the farming sector include the management of agricultural pesticides (Beck et al., 1989), farm financial planning (Boggess et al., 1989, Herrero et al., 1996), livestock enterprises (Stuth and Lyons, 1993), and land and crop nutrient planning (Basso et al., 2013, Quemada and Cabrera, 1995, Li et al., 2009), and river basin management Semenzin et al. (2012). DSSs have mainly been designed to support farm advisors and other involved technicians in their interaction with policy makers and farmers (Nelson et al., 2002), although some systems allow direct use by the latter. In addition to this type of farm-level decision making support, agricultural system models are being increasingly used for various local, national and global modeling tasks and analyses (Jones et al., 2017).

Compared to other DSSs used in agriculture, whose focus is the simulation of different scenarios against various management alternatives (Jones et al., 2003, McCown et al., 1996), our proposal integrates simulation modules and optimization algorithms. It seeks to identify efficient management alternatives to hypothetical agricultural scenarios, as e.g. population growth, climate change or increase in system inputs. Motivated by the context of the Mékrou project, where highly committed local stakeholders conveyed their actual needs straightforwardly, our objective was to develop an integrated modelization-optimization-easy-to-use DSS for the management of the Mékrou basin, aiming to reduce poverty and increase growth in the region.

The remainder of the paper is organized as follows. In Section 2, we describe the DSS components. The Mékrou region case study is introduced in Section 3, for which some DSS capabilities are illustrated in Section 4. We end up in Section 5 with a discussion of our main findings and future lines of research.

## 2 Methodology

Our aim is to assess how local agricultural production may potentially mitigate food insecurity. In doing so, we need to deal with the following issues: (1) multiple demands of spatially distributed food crops; (2) limited fertilizers and water availability; and (3) variable crop distribution and productivity along the river basin.

**Fig. 1:**
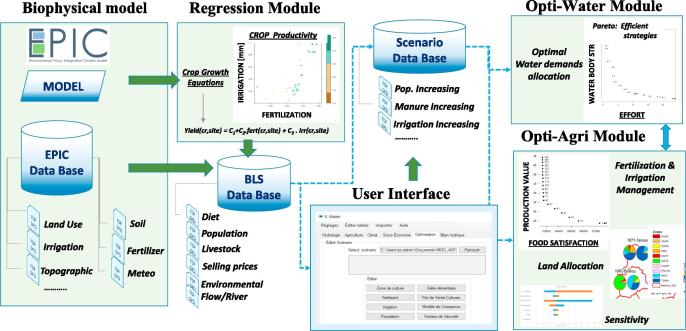
Schematic representation of our DSS.

To that purpose, a DSS based on a built-in baseline scenario was developed, integrating several models, tools and data, see Fig. 1: (1) A geodatabase storing the required data to model (soil type, climate, landuse and landcover), and to assess economic and food security (based on crop market selling prices, crop use, productivity and management, diet habits and minimum food calories intake requirements). (2) A biophysical crop-growing model, able to simulate multiple crop yield at local level under different environmental and management conditions. (3) A simplified multiple linear regression metamodel, derived from the crop model outputs, used to estimate yield production for each crop and region in terms of the applied fertilizer and irrigation. (4) A linear programming optimization routine, incorporating the local crop growth metamodel outputs, the available fertilizer and the irrigation water, and considering as decision variables the total crop area and distribution. (5) A multiobjective genetic algorithm routine, designed to spatially optimize water demands—subject to its availability across the river basin—from different sectors, as e.g. agricultural, urban or livestock.

In what follows, we describe the most complex modules in detail.

### 2.1 The biophysical model

EPIC is a biophysical, continuous, field-scale agriculture management model (Williams et al., 1989, Williams, 1995). It simulates crop growth, crop water requirements and the fate of nutrients and pesticides as affected by farm management activities, such as the timing of agrochemicals application, tillage, crop rotation, irrigation strategies, and nitrogen and phosphorus cycle.

The main components can be divided into the following categories: hydrology, weather, erosion, nutrients, and plant growth. EPIC estimates the development of crop growth on a daily time-step basis, taking light interception and conversion of CO_2_ to biomass into account. In turn, the phenological development is based on daily heat units accumulation (PHU, 2007). As regards crop yield, it is calculated, *via* harvest index, as a fraction of above-ground biomass, and reduced according to daily water and nutrient stress. On the other hand, the hydrological model stems from the water balance equation in the soil profile, with several processes simulating surface runoff and infiltration, evapotranspiration, lateral subsurface flow, and percolation. Finally, EPIC takes nitrogen and phosphorus cycles (mineralization, denitrification, volatilization and fixation processes) into account. For a detailed description of the model and the simulated processes see Williams, 1995, Wang et al., 2012, Pastori et al., 2017.

In our case study, EPIC was applied to the dominant crops in each grid cell (3 km resolution) in the period between 1990 and 2016. Specifically, the crops analyzed in this study were: (1) cereals (maize, sorghum, millet and rice); (2) tubers (cassava, yam, and potatoes); (3) leguminous (a vegetable crop, cowpea and soybeans); (4) fruit (peanuts, oils, banana, although the latter is not spread in the region); and (5) a cash crop (cotton).

### 2.2 The regression module

In order to involve stakeholders effectively in participatory modeling tasks, simplified models allowing them to make informed and timely decisions are required (Soltani et al., 2013). Such models must, however, be endowed with a realistic sensitivity to the relevant input parameters (Collins et al., 2013). As mentioned before, we have firstly used EPIC to simulate the crop growth in the Mékrou river basin. However, instead of the built-in EPIC model, we have integrated an *ad hoc* metamodel within the optimization tool for computational reasons. Such choice represents a compromise between admissible simulation times and the ability to take relevant relationships—as e.g. linking crop growth behavior to management strategies and environmental conditions—into account (Barton, 1998, Kleijnen and Sargent, 2000). In our case, we have considered a simple multiple linear regression model, mimicking the input-output relationships of the complex process-based EPIC model. The response variable is the crop yield, whereas the fertilizer and irrigation applied are considered as explanatory variables. Integrated within a DSS, the metamodel provides well-timed solutions, although the computational burden of detecting optimal management patterns can sometimes be significant.

In order to address spatial environmental variability (including physical characteristics potentially affecting the crop growing process, as e.g. soil suitability, climate or rain), specific metamodels were devised for each crop and region sharing similar soil and climate conditions. Under this approach, the influence of the environmental conditions on the crop yield can be regarded as homogeneous at the administrative level defined by regional communes. Since most agricultural strategies and policy making actions are generally defined and adopted at this spatial level, that local scale seems a reasonable choice. Finally, we also took the potential variability of crop production under different management strategies into account, considering alternative scenarios to the baseline one, derived from current management practices.

### 2.3 The optimization module: model formulation and objectives

Our tool includes two optimization routines, denoted by Opti-Agri and Opti-Water in Fig. 1. Opti-Agri focuses on the optimal management of agricultural crops related to food security (the so-called food crop demand satisfaction), and on the efficient use of limited resources (fertilizers, water and land). The solver included in this routine is based on linear programming techniques. In turn, Opti-Water identifies efficient water management strategies, spatially optimizing conflicting water demands arising from various sectors along the river basin.The solver included in this routine has a genetic algorithm nature. Note that both modules are linked, since the agricultural water demand feeding the Opti-Water model is actually an output of the Opti-Agri model. We describe them more in detail in the next section, outlining the relevant notation in Table 1.

**Table 1:** Notation used in the optimization models.

Subscripts	
*r*	Region
*c*	Crop
*cg*	Crop group
*sb*	Sub-catchment
*R*	All regions
Decision variables	
${\mathrm{Fert}}_{r,c}$	Fertilization rate
${\mathrm{Irr}}_{r,c}$	Irrigation rate
${\mathrm{Area}}_{r,c}$	Agricultural area
${\mathrm{DR}}_{\mathrm{sb}}^{U}$	Reduction of urban demand
${\mathrm{DR}}_{\mathrm{sb}}^{A}$	Reduction of agricultural demand
${\mathrm{DR}}_{\mathrm{sb}}^{L}$	Reduction of livestock demand
Parameters	
${\mathrm{FRD}}_{r,c}$	Food requirements deficit
${\mathrm{APS}}_{r,c}$	Agricultural production “surplus”
${\mathrm{SP}}_{r,c}$	Selling price of crops
${\mathrm{AP}}_{r,c}$	Agricultural production
${\mathrm{FR}}_{r,c}$	Food requirement
${\mathrm{FRI}}_{r,\mathrm{cg}}$	Individual food requirement
$P_{r}$	Total population
${\mathrm{CFixProduct}}_{r,c}$	Intercept of crop growth regression model
${\mathrm{CGrowF}}_{r,c}$	Fertilization coefficient of crop growth regression model
${\mathrm{CGrowI}}_{r,c}$	Irrigation coefficient of crop growth regression model
${\mathrm{MaxLimFert}}_{r,c}$	Maximum fertilizer
${\mathrm{MinLimFert}}_{r,c}$	Minimum fertilizer
${\mathrm{MaxLimIrr}}_{r,c}$	Maximum irrigation
${\mathrm{MinLimIrr}}_{r,c}$	Minimum irrigation
${\mathrm{TopAreaReg}}_{r}$	maximum agricultural area
${\mathrm{RatMaxReg}}_{r,c}$	Maximum proportion of area available
${\mathrm{WBS}}_{\mathrm{sb}}$	Water body status
${\mathrm{WEI}}_{\mathrm{sb}}$	Water exploitation index
${\mathrm{TD}}_{\mathrm{sb}}^{U}$	Total urban demand
${\mathrm{TD}}_{\mathrm{sb}}^{A}$	Total agricultural demand
${\mathrm{TD}}_{\mathrm{sb}}^{L}$	Total livestock demand
$W^{U}$	cost/m^3^ of reducing urban consumption
$W^{A}$	cost/m^3^ of reducing agricultural consumption
$W^{L}$	cost/m^3^ of reducing livestock consumption

#### 2.3.1 Opti-Agri module

It considers two main goals: (1) An economic objective, aimed at maximizing farmers’ total benefit; and (2) A social objective, trying to minimize food self-sufficiency. To tackle their simultaneous optimization, we define auxiliary objective functions:

1. Minimize the deficit of population food requirements
  $$\min\underset{r}{\sum }\underset{c}{\sum }{\mathrm{FRD}}_{r,c}$$
2. Maximize the benefit of total agricultural excess
  (1) $$\max\underset{r}{\sum }\underset{c}{\sum }{\mathrm{APS}}_{r,c}\cdot {\mathrm{SP}}_{r,c}$$

The production dedicated to satisfy population food requirements does not result into economic benefits. However, both objectives are actually not entirely conflicting, since greater productions could eventually help reduce the deficit of food requirements, while increasing, at the same time, farmers’ benefit. Indeed, it may sometimes be impossible to fully satisfy food demand, in which case the optimizer would not return a solution. To avoid this eventuality, we reformulate (1), considering food demand as a constraint, and including a slack variable to guarantee the existence of a feasible solution:

$$\begin{array}{cc} \max & \underset{r}{\sum }\underset{c}{\sum }({\mathrm{APS}}_{r,c}\cdot {\mathrm{SP}}_{r,c}-\log f_{r,c})\\ \text{s.t.} & {\mathrm{FRD}}_{r,c}+\log f_{r,c}⩾0 \end{array}$$

*APS* and *FRD* can actually be defined in terms of the agricultural production and the food requirement as follows:

$$\begin{array}{cc} & {\mathrm{APS}}_{r,c}=\left\{\begin{array}{cc} {\mathrm{AP}}_{r,c}-{\mathrm{FR}}_{r,c} & \text{if}\;{\mathrm{AP}}_{r,c}⩾{\mathrm{FR}}_{r,c}\\ 0 & \text{otherwise}, \end{array}\right)\\ & {\mathrm{FRD}}_{r,c}=\left\{\begin{array}{cc} {\mathrm{FR}}_{r,c}-{\mathrm{AP}}_{r,c} & \text{if}{\mathrm{AP}}_{r,c}<{\mathrm{FR}}_{r,c}\\ 0 & \text{otherwise}, \end{array}\right) \end{array}$$

being *FR* a function of the diet and the population size

$${\mathrm{FR}}_{r,c}={\mathrm{FRI}}_{r,c}\cdot P_{r}$$

In turn, *AP* is a linear function of the fertilization and irrigation rates

$${\mathrm{AP}}_{r,c}={\mathrm{CFixProduct}}_{r,c}+{\mathrm{CGrowF}}_{r,c}\cdot {\mathrm{Area}}_{r,c}\cdot {\mathrm{Fert}}_{r,c}+{\mathrm{CGrowI}}_{r,c}\cdot {\mathrm{Area}}_{r,c}\cdot {\mathrm{Irr}}_{r,c}$$

The decision variables are ${\mathrm{Fert}}_{r,c},{\mathrm{Irr}}_{r,c}$ and ${\mathrm{Area}}_{r,c}$, and they account for the different management options of fertilizer, irrigation and agricultural land distribution. The first two are limited by the following bound constraints:

$$\begin{array}{cc} & {\mathrm{MinLimFert}}_{r,c}⩽{\mathrm{Fert}}_{r,c}⩽{\mathrm{MaxLimFert}}_{r,c}\\ & {\mathrm{MinLimIrr}}_{r,c}⩽{\mathrm{Irr}}_{r,c}⩽{\mathrm{MaxLimIrr}}_{r,c} \end{array}$$

Besides, we set constraints for the total fertilizer and irrigation available at regional, country or global scale:

$$\begin{array}{cc} & \underset{c}{\sum }{\mathrm{Fert}}_{r,c}\cdot {\mathrm{Area}}_{r,c}⩽{\mathrm{FertAvail}}_{r}\\ & \underset{c}{\sum }{\mathrm{Irr}}_{r,c}\cdot {\mathrm{Area}}_{r,c}⩽{\mathrm{IrrAvail}}_{r} \end{array}$$

We also define lower and upper bounds for the area dedicated to each crop

$${\mathrm{AreaMin}}_{r,c}⩽{\mathrm{Area}}_{r,c}⩽{\mathrm{AreaMax}}_{r,c}$$

Similarly, the total agricultural area available is bounded above by

$$\underset{c}{\sum }{\mathrm{Area}}_{r,c}⩽{\mathrm{TotAreaReg}}_{r}\;\forall \;r\;\mathrm{in}\;R$$

Finally, the maximum proportion of area available for a single crop in the same region is given by

$${\mathrm{Area}}_{r,c}⩽{\mathrm{RatMaxReg}}_{r,c}\cdot {\mathrm{TotAreaReg}}_{r}$$

#### 2.3.2 Opti-Water module

It is aimed at reducing the impact on water usage in the region. It also helps to identify the main stresses that could potentially affect water resources in the river basin, as well as the efforts that would be necessary to implement to enhance its usage. The solutions provided by the system are site- and sector-specific, i.e., adapted to each region and demanding activity.

Taking both environmental and economical considerations into account, this is a multiobjective problem, with the following objective functions:

1. Maximize the water body status (flow regime)
  (2) $$\max\underset{\mathrm{sb}}{\sum }{\mathrm{WBS}}_{\mathrm{sb}}$$
  As a measure of the water body status, we use the water exploitation index (WEI), defined as the ratio of withdrawals from renewable natural water resources to average renewable natural water resources (Alcamo et al., 2017, Rijsberman, 2006).
  (3) $${\mathrm{WEI}}_{\mathrm{sb}}=\frac{{\mathrm{TD}}_{\mathrm{sb}}^{U}+{\mathrm{TD}}_{\mathrm{sb}}^{A}+{\mathrm{TD}}_{\mathrm{sb}}^{L}}{{\mathrm{Naturalflow}}_{\mathrm{sb}}}$$
  Therefore, we can be reformulate (2) in terms of *WEI* as
  $$\min\underset{\mathrm{sb}}{\sum }{\mathrm{WEI}}_{\mathrm{sb}}$$
  However, minimizing *WEI* requires an effective decrease in the demand, whose effect on the status of the basin depends on the actual point of application. But applying the same reduction to each point in the basin is not an easy task, and it is also greatly influenced by the water usage (Pistocchi et al., 2017). Therefore, we can express the economic objective as:
2. Minimize the effort required (water demand reduction)
  $$\min\underset{\mathrm{sb}}{\sum }({\mathrm{TD}}_{\mathrm{sb}}^{U}\cdot {\mathrm{DR}}_{\mathrm{sb}}^{U})\cdot W^{U}+({\mathrm{TD}}_{\mathrm{sb}}^{A}\cdot {\mathrm{DR}}_{\mathrm{sb}}^{A})\cdot W^{A}+({\mathrm{TD}}_{\mathrm{sb}}^{L}\cdot {\mathrm{DR}}_{\mathrm{sb}}^{L})\cdot W^{L}$$

#### 2.3.3 Optimization solvers

To solve the Opti-Agri linear programming model, we relied on the software package GLPK (Makhorin, 2012), licensed under the GNU General Public License. Since the DSS core was developed in R (R Core Team, 2017), we used the Rglpk (Theussl and Hornik, 2017) package to provide a high level solver function based on the low level C interface of the GLPK solver. The modules of the agronomic optimizer were coded in GNU MathProg, which is a modification of AMPL (Fourer et al., 1990). For the Opti-Water optimizer, the NSGA II multiobjective genetic algorithm (Deb et al., 2002, Udias et al., 2018) was applied, using its R implementation, nsga2R (Tsou, 2013).

## 3 A case study

**Fig. 2:**
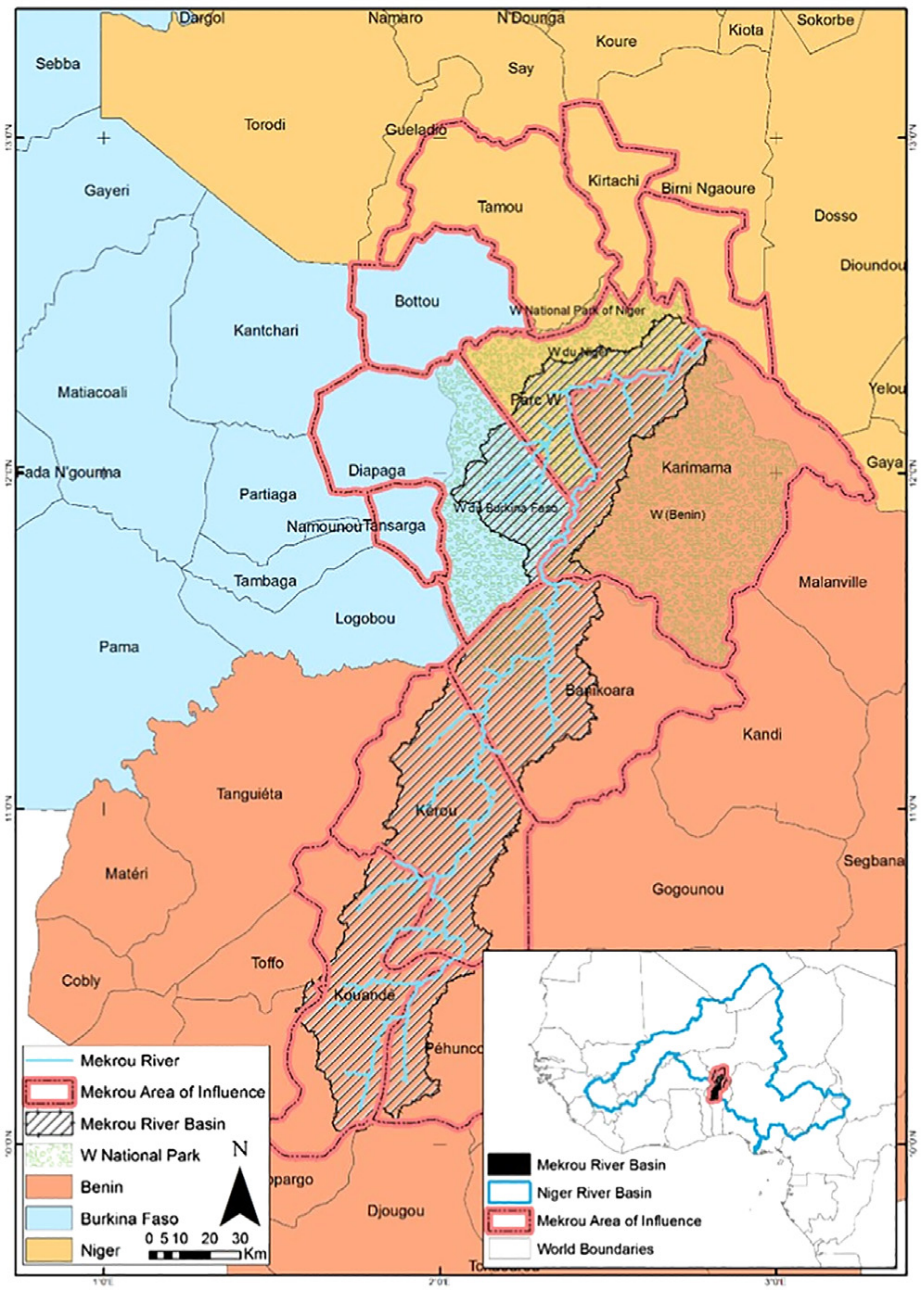
Location of the Mékrou river basin.

The transboundary Mékrou river basin—representing 3% of the wider Niger river basin area—covers an area of 10 635 km^2^, see Fig. 2. Running across Benin (80%), Burkina Faso (10%) and Niger (10%), it is characterized by underdeveloped infrastructures and poor socioeconomic conditions. Agriculture is indeed the key economic sector for poverty alleviation and food security in the three riparian countries. Arable land accounts for 22% of total area, and it is mainly used for food crop production and raising cattle. The current extension of cultivated area in the region is approximately 730 000 ha.

The water resources of the Mékrou river have several uses, being the most representative ones domestic consumption, crop irrigation, animal production, fishing and fish farming, recreation and religious practices. The total need for drinkable water in 2014 was estimated at 1 957 358 m^3^ and 1 148 884 m^3^ in urban and rural areas, respectively, serving a population of 280 000 inhabitants. The main pressures on water resources come from production activities related to agriculture, livestock, fisheries, forests, hunting, mining, industry and energy.

The Mékrou river basin includes five Communes in Benin (Banikoara, Karimama, Kérou, Kouandé and Péhunco), two in Niger (Kirtachi and Parc W) and another one in Burkina Faso (Diapaga). However, in addition to the hydrological definition of the Mékrou river basin as the Area of Influence on the local economy, we also include the so-called Area of Interest, which comprises another two Communes in Burkina Faso (Tansarga and Bottou), and Niger (Tamou and Birni Ngaoure). The population of the twelve Communes is shown in Table 2.

**Table 2:** Mékrou Communes and their estimated population (2016).

Country	Identifier	Commune	Source	Population
Benin	1062	Banikoara	INSAE, 2013	284 313
	1063	Karimama	INSAE, 2013	76 866
	1064	Kérou	INSAE, 2013	111 180
	1065	Kouandé	INSAE, 2013	122 675
	1066	Péhunco	INSAE, 2013	86 005

Burkina Faso	1067	Bottou	ISND 2006	68 020
	1068	Diapaga	ISND 2006	48 965
	1069	Tansarga	ISND 2006	56 549

Niger	1070	Kirtachi	INS 2011	39 133
	1071	Tamou	INS 2014	95 527
	1072	Birni Ngaoure	INS 2014	78 000
	1073	Parc W	INS 2014	0

**Total**				1 067 232

In the current situation, dry cereals (sorghum, millet, maize and rice), tubers (mainly yams and cassava), oilcrops (sesame), legume (cowpea) and cash crops (mainly cotton) are all rainfed crops sown during the rainy season (April-May to late September early October), therefore not receiving extra water input from irrigation. In turn, other crops (mainly vegetables and rice, which is used both in rainy and dry seasons) demand irrigation and, therefore, will depend on, and need to be close to, shallow water resources from small ponds and rivers.

### 3.1 Source of data

#### 3.1.1 EPIC modeling

A geodatabase was developed to support the application of EPIC to the area of study. It stores the data required by: (i) EPIC (including soil, meteorological daily data, crop distribution and agriculture management information) to simulate different agronomic management strategies; and (ii) the optimization analysis tool (including population density, per capita food calories requirements, diet habits, total food demand, expected trend of population growth and selling prices of agricultural products).

Specifically, the following datasets were used to setup EPIC: (1) The Harmonized World Soil Database (HWSD) (FAO, 2017b) with an approximate resolution of 1 km (30″) was used to characterize the soils of the simulation units. (2) A global digital elevation model with a horizontal grid spacing of 30″ (NASA, 2017) providing the elevation and slope. (3) Daily meteorological data (including precipitation, wet-day frequency, minimum and maximum temperature, relative humidity, solar radiation, and wind speed) with a 10′ resolution derived from the ERA-Interim global dataset (Berrisford et al., 2009). It resulted in 125 virtual stations in the area of study for the period 1990–2012. (4) A global crop distribution dataset derived from the Spatial Production Allocation Model, SPAM (You et al., 2017), used to calculate the share of each crop for each EPIC simulation unit.

Crop management is one of the most important inputs required for EPIC modeling. It consists of detailed schedules and features of the most common crop operations—like e.g. sowing, harvesting, tillage timing, mineral fertilization, manure usage or irrigation—for each crop used in the region. In this study, crop management practices were considered reasonably homogeneous at the administrative level (communes), meaning that just one strategy will be applied to each commune. In general, all crop operations have been defined at this level, although more specific fertilization and/or irrigation strategies could be established for finer resolutions if required.

Given the fact that the dominant agriculture is rainfed, rain is the most liming factor controlling farmers’ strategies. Therefore, crops sowing periods were designed based on precipitation patterns, coinciding with the start of the rainy season, following the method devised by Rojas et al. (2005) and described in more detail in Pastori et al. (2011). The growing period length and the harvest timing were based on air temperature and the PHU accumulation method (Rijsberman, 2006).

The most recent version of the global map of irrigated areas from FAO was used as the main reference to identify those areas where irrigation needed to be considered (FAO, 2018b). According to it, only some areas along the Niger river are actually using irrigation water, something confirmed by the analysis of a household survey (Markantonis et al., 2017b), where only Nigerian farmers reported significant use of irrigation as a normal practice. In turn, nutrients input from manure was derived from the livestock density (Robinson et al., 2014), considering different coefficients for each animal category to calculate the actual quantity of available nitrogen for crops. Finally, mineral fertilization was obtained from national statistics reporting total use of nitrogen for each crop (FAO, 2017a), and assigned to specific crops according to information coming from local surveys and current yield productivity.

#### 3.1.2 Multiobjetcive optimization

In order to perform the DSS optimization analysis, specific data, defined at the administrative scale units, were required. The total production under current conditions was extracted from national statistics in 2016, see Table 3.^1^ Population density and growth rates were also obtained from regional statistics reported by the different countries.

**Table 3:** 2016 production (tons) by crop and region.

Id.	Commune	BANA	CASS	CORN	COTS	COWP	PMIL	PNUT	POTA	RICE	SGHY	TOMA	YAMS
1062	Banikoara	2348	11 546	48 992	56 788	16 500	455	3427	50 734	9575	24 579	3111	49 479
1063	Karimama	7485	178	11 437	1102	6165	4512	144	309	22 243	6017	17 355	1030
1064	Kérou	2833	298	6078	26 912	266	21	12 857	173	120	682	11 214	25 420
1065	Kouandé	3134	80 157	39 339	13 829	2248	324	36 145	263 167	8187	8506	25 502	225 253
1066	Péhunco	530	36 975	31 919	12 210	450	217	9090	2098	3275	4319	4927	63 628
1067	Bottou	1062	0	16 409	13 588	850	2861	4222	561	3260	21 970	7623	65
1068	Diapaga	320	41	10 152	7053	363	1228	1929	283	3616	11 936	6726	98
1069	Tansarga	381	36	6816	3858	217	927	1709	382	803	12 859	6770	44
1070	Kirtachi	1662	0	7326	0	219	20 677	466	17 002	4278	8053	12 180	1984
1071	Tamou	4889	0	15 294	147	524	51 532	1114	31	10 183	19 040	30 184	4
1072	Birni Ngaoure	13 294	10	7338	7	997	44 091	1070	0	6342	7762	3804	331
1073	Parc W	0	0	0	0	0	0	0	0	0	0	0	0

Food demand was calculated by combining diet habits of local population with the average dietary energy requirement (ADER) as defined by FAO (FAO et al., 2014, FAO, 2018a) The latter was estimated at 2400 kcal/pers/day for the three countries. Diet habits were, in turn, gathered from local surveys (put in place purposely in each country for the Mékrou project), and based on the annual per capita quantity of each food crop consumed. This modified safe diet has the advantages of taking the preferences of local population into account, guaranteeing, at the same time, a security condition for food intake. Nevertheless, there are other aspects that could potentially affect food crop insecurity, such as post-harvest losses, limited accessibility to market, lack of infrastructure for food transport and storage, and a cropping system highly sensitive to local and seasonal conditions. In order to incorporate all these issues into the analysis of food production, we used a food security factor, measuring the effective quantity of food available for consumption, ranging from 20% for most crops to 50% for rice and oil crops.

Finally, the economic value of agricultural production was also addressed, by considering selling prices—as reported from local surveys—for crop items produced in the area under study.

## 4 Analysis of results (DSS application)

In this section, we present the application of our DSS. Our main aim is to forecast food demand by 2025, assuming current diet habits, a total food calories intake of 2400 kcal/day and an annual 3% increase in population, see Table 3. We derived specific equations to assess the influence of management strategies and climate variability on food crop growth, from a long-term simulation covering the period 1990–2016. Local agricultural production in the region includes several annual and perennial crops, being the most relevant ones: cereals (maize, millet, sorghum and rice), tubers (yams and cassava). Less spread, but important especially for food diet, are potatoes, legumes with the dominance of cowpea and peanuts and vegetables. Additionally, we also simulated some tropical fruit crops and the cash crop cotton, a primary source for the local agricultural economy, especially in Benin.

A flag variable, linked to local food production and demand, was introduced to assess the potential risk associated with food safety. Should food demand of a certain item not be satisfied with local production, the flag variable would be set to “missing”, indicating infeasibility.

**Fig. 3:**
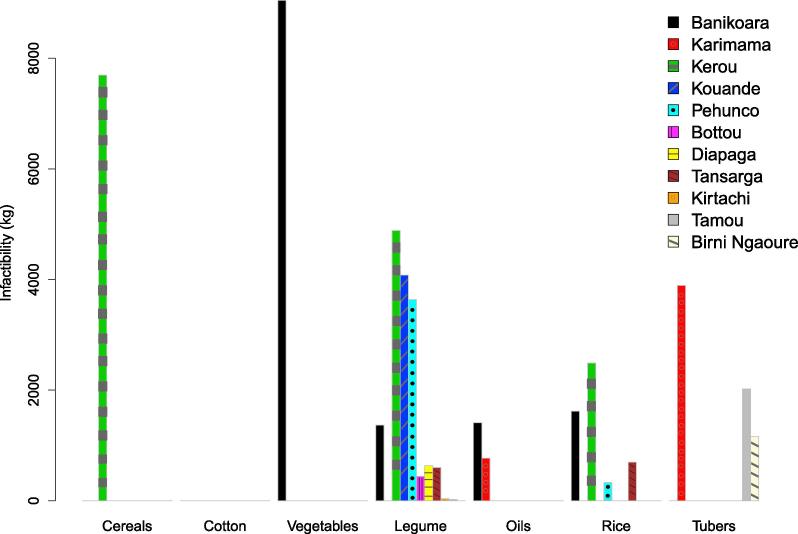
Infeasibility (food self-sufficiency indicator) by crop and region for Baseline 2025 (BLS_2025 as defined in Table 4).

The DSS output is analyzed by means of graphics or tables, highlighting, for instance, eventual missing quantities for each main crop food item at the regional level. Results for the analysis of the 2025 food demand scenario are shown in Fig. 3. It is important to stress that this indicator of food infeasibility is strictly linked to local production capacity, because it is not possible to exchange food items between different countries and regions.

In our case study, we assume that food crops cannot be traded between regions or countries, since our goal was to assess the self-sufficiency of local agriculture production. Besides, most food crop production is provided by small farmers, who aim to cover their own subsistence, or to sell the excess in local markets. In addition, limited road infrastructures and poor market organization would tend to further decrease the possibility of such exchange. Nevertheless, some of these alimentary infeasibilities would disappear globally at the level of Area of Influence. Indeed, food crop demand is higher than production in some regions (violating food security), whereas in others there is a surplus of production, resulting in a net economic benefit for farmers. When local food demand is fully satisfied (overproduction), all the exceeding quantities are accounted for to calculate the additional economic value. This is done based solely on selling prices, while disregarding costs related to fertilizer and irrigation management.

### 4.1 Validation of the metamodel

**Fig. 4:**
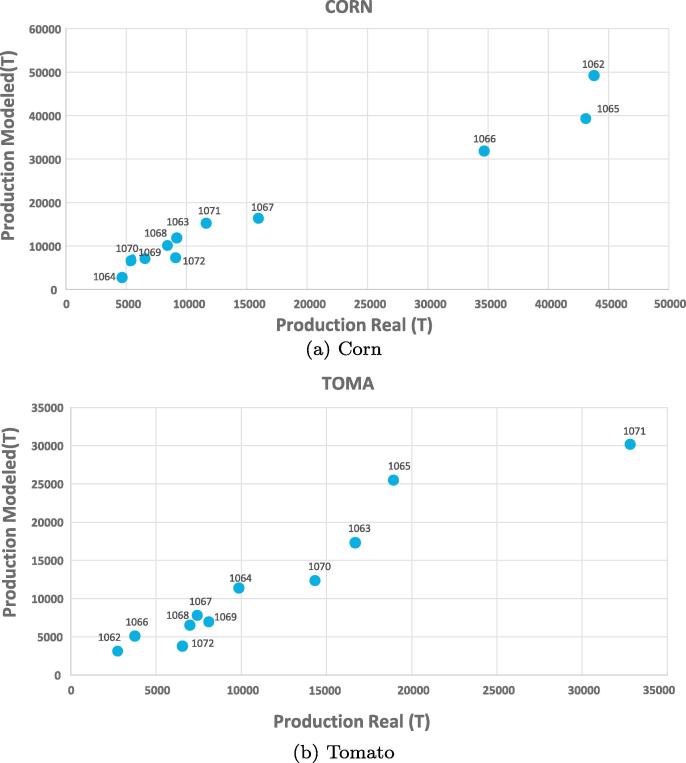
Real vs modeled production for corn and tomato by region.

Results provided by the decision tool are significantly affected by the accuracy of the agricultural production simulation module. Therefore, we need to verify the local prediction capacity of the embedded regression models. Although we found slight variations for different crops and regions, correlations between real and modeled values were, in general, above 0.7, suggesting a good fit. The validation was performed at the regional level (numbers in the Figure refers to different regions) by comparing the average predicted and reported yields. The comparison for two dominant crops (corn for cereals and tomato for vegetables) is given in Fig. 4. The simulated and the reported yields compare well and the model is able to capture the production variability across the simulated regions.

### 4.2 Water availability

This module was run to shed light over the compromise between the water used for different purposes (irrigation, livestock, urban) and the environmental state of the river, evaluated through the WEI index (3). Both objectives are conflicting, since extracting too much water from the basin could jeopardize its environmental state.

**Fig. 5:**
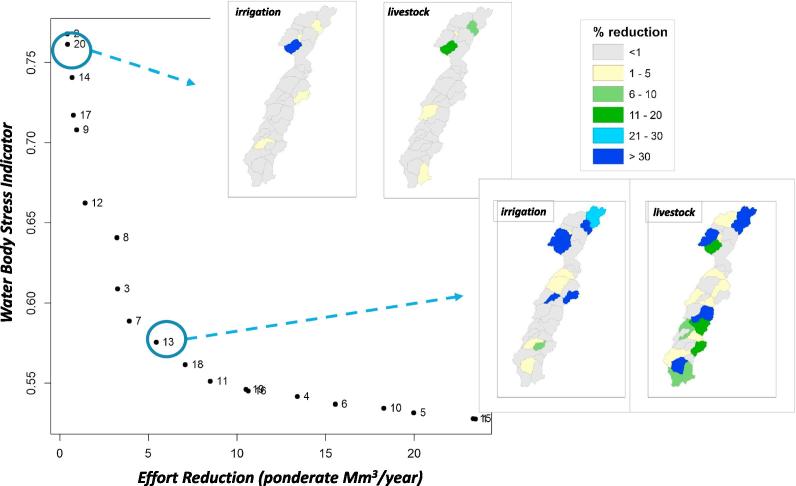
Efficient Pareto water demand reduction according to water body stress indicator (third quartile) and the sum of demand reductions.

The tool allows to identify, for each region, which strategies of demand reduction produce a greater decrease in the impact on the natural river flow. WEI can be assessed using one of the following aggregation metrics: max, quantile, threshold or mean. Specifically for the results showed in Fig. 5, we used the third quartile of WEI as the aggregation metric. In addition, the optimization problem can be launched configuring the relative importance of each water demand usage compared with others. Therefore, users will be able to assess the different costs required to reach the same benefit for each water demand. To wit, in our case study we considered the implementation effort of reducing livestock and urban demands twice and ten times greater than decreasing irrigation demand, respectively.

Fig. 5 shows the Pareto frontier, formed by the trade-off efficient strategies between the WEI and the costs of demand reduction. As we can observe, the former can be significantly decreased by reducing water detractions from the river basin. In order to show how different reductions in basin pressure can affect the global water body stress we have selected two strategies. Strategy #20 is similar to the one in force, with a stress index over 75%, and a very limited reduction in demand applied. After implementing it, pressures were slightly reduced only at four sub-basins, among which only one experimented a drop-off in the consumption for irrigation greater than 30%. On the other hand, strategy #13 achieved a significant reduction in WEI close to 55%, with several decreases in consumption for irrigation and agriculture. More extreme strategies rightward of #13 obtained considerably larger improvements in the river status (around 53%, almost twice bigger than that of #13), but at the cost of almost four times greater efforts.

### 4.3 Crop management (land allocation, fertilization, irrigation)

As we have already mentioned, crop production is a key issue in the Mékrou area for local economy and food security. With the aid of our DSS, we were able to assess the ability of the agriculture sector to satisfy the food demand of local population, subject to different agricultural management strategies and external constraints. Thus, we were able to identify site-specific optimal planning for efficient land management, combined with enhanced fertilization/irrigation strategies, and taking other constraints—as e.g. population density, minimum dietary requirement and habits, and crop selling prices—into account.

Different scenarios, corresponding to various strategies for land and crop management depending on changes in external constraints, were tested and compared with a baseline setting, see Table 4. This baseline scenario (BLS) referred to current conditions and management in the Mékrou river basin. A second baseline scenario (BLS_2025) was defined to analyze the 2025 time horizon, including new constraints and production targets. It is based on the expected annual 3% population growth rate, on local diet information (as extracted from local surveys), and on the FAO standards for the required daily intake (2400 kcal/day/hab). As we can observe, current food infeasibility is expected to almost double by 2025, while the total gross benefit (without introducing new management strategies) would significantly reduce 16%.

**Table 4:** Total estimated benefit and infeasibility for different scenarios.

Scenario	Description	Benefit (€)	Infeas. (kg)	Infeas. (€)
BLS	Current condition 2016	179 457	46 864	22 591
BLS2025	Food demand increase 2025	154 583	91 041	43 838
BLS2025_F200_I200	Fert. and irrig. increase + 200% (vs BLS)	325 468	65 410	31 217
Cotton0to100	Cotton land redistribution	437 720	15 178	3609
Rest 0to100	Free cropland redistribution	396 775	7230	3376
BLS2025_10_60	Constrained cropland redistribution	487 146	3720	970

**Fig. 6:**
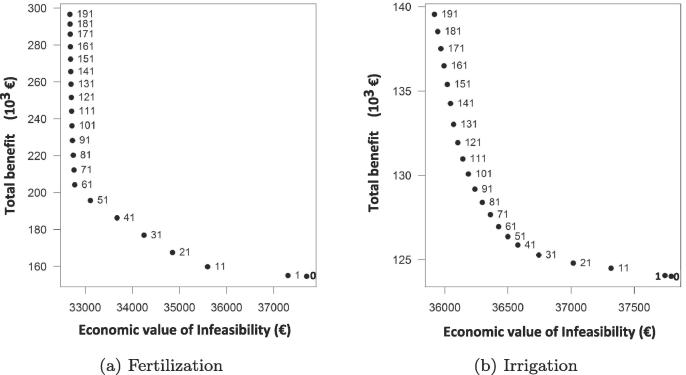
Total benefit from the selling crop surplus and economic value of food security infeasibility when BLS_2025 fertilization (left) and irrigation (right) conditions increase. Number labels represent the increasing rate.

Alternative management strategies were also included in the analysis. For instance, we analyzed the eventual satisfaction of local food self-sufficiency by increasing the intensity and efficiency of irrigation and fertilization—currently very low compared with world standards—and introducing different cropland allocation strategies. Results refer to the 2025 scenario, see Fig. 6, since its associated increase in food production infeasibility is particularly useful to explore our ability to identify more intensive and efficient alternative solutions.

#### 4.3.1 Fertilizers

The tool tried different solutions, aiming to redistribute fertilizer and irrigation water in an optimal way for each crop and region, although country and global options were also available. As expected, the impact of using additional nutrients in reducing food demand becomes evident when comparing it with sole irrigation practices, due to: (i) a very low current level of fertilization, with crop production mainly limited by nutrient fertility; and (ii) the standard growing period taking place during the rainy season, when the importance of irrigation can become crucial (especially in dry years, or when dry spells occur at the start and/or end of vegetative seasons). In fact, it can be observed that the influence of fertilization on the benefit is almost twice as strong as the irrigation effect. It seems that further increments in either fertilizer or irrigation would still produce additional increases of benefit. Note, however, that even when combining both strategies, some important infeasibilities still remain. To wit, using 60% more fertilizer than in current practice does not significantly reduce the satisfaction of food demand, implying that the extra fertilizer impacts only on the benefit, see Fig. 6a. Considering, in turn, the impact of irrigation on benefit and demand, we observe that, even when doubling the water used for irrigation, the Pareto curve does not reach its asymptotic value, see Fig. 6b.

**Fig. 7:**
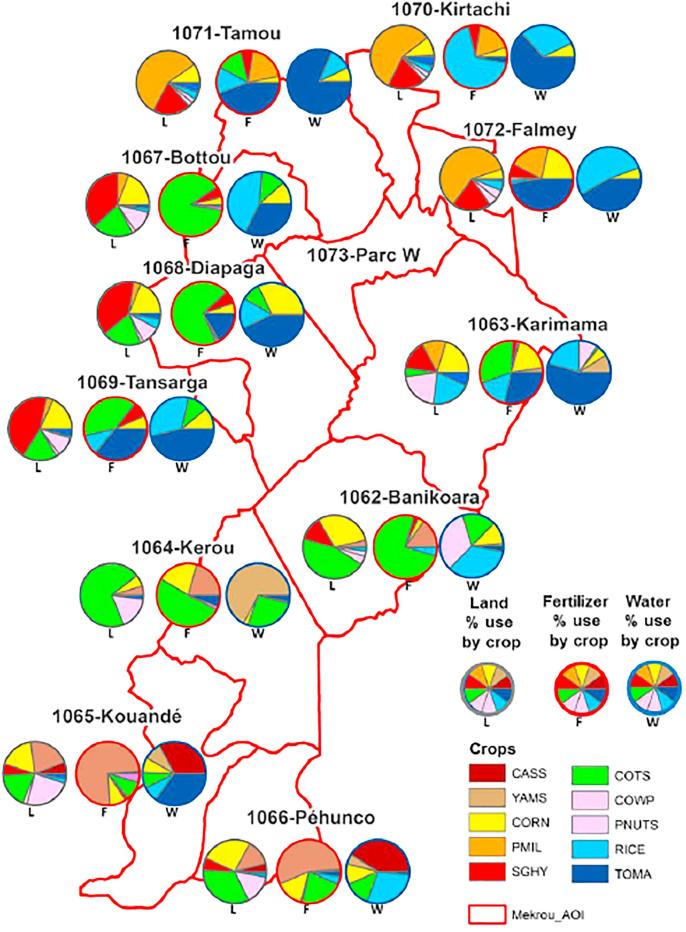
Crop regional distribution (baseline surfaces), and fertilization and irrigation proportions under BLS_2025_FI200 scenario. From left to right, the three piecharts represent the shares of: cropland occupied (L), total fertilizer (F) and water (W) for each crop and region.

Based on the previous analysis, we have considered an alternative scenario BLS_2025_FI200, see Table 4, where irrigation and fertilization are simultaneously increased by 200%. Fig. 7 shows the proportion of both resources used in each crop and each region under such a scenario. The values represented refer to those quantities of fertilizer and irrigation water identified as efficient strategies, which depend on the application rate, but also on the effective surface used for each crop in the region.

We focus now on cotton, which is an important nonfood crop, intensively used in the area of study, particularly in Benin and Burkina Faso. Since it is a cash crop, it attracts the biggest share of fertilization (around 57%) under current management strategies. In this sense, it is interesting to note that, even by including the food safety constraint (the dominant objective is to satisfy local food demand), cotton (a nonfood crop) is still the most fertilized crop. This is explained by the fact that changes in landuse are not allowed in this scenario, and, therefore, cotton remains the dominant crop for land occupation. Furthermore, when food crops produce no additional income, farmers can increase their benefit by fertilizing cotton. Under this scenario, the proposed fertilization strategy for cotton is considerably intensive, corresponding to an average use of around 150 kgN/(ha·yr), compared with the baseline value of 40.

Among food crops, the distribution of nutrients varies considerably for each region and country. In the Benin area, most fertilization under current management practices (BLSs scenarios) is used for maize and tubers, and locally also for rice. On the other hand, fertilization is very low in the Niger area, with values within the range 1–2 kgN/(ha·yr), implying that, in practice, crops would only receive nutrients input when livestock manure is available. Since crop distribution is not altered under the optimized scenario, the resulting quantities of fertilizers used by crop depend exclusively on their own cropland occupation. Thus, most fertilization is used for tubers and maize, and a smaller share for vegetables and rice. Tubers are indeed a highly intensive crop, since: (i) their productivity is enhanced by increasing fertilizers; (ii) they are required in Benin for food demand satisfaction; and (iii) their economic benefit also boosts as the average fertilization rate rises from 20 to around 400 kgN/(ha·yr).

As regards vegetables, apart from being needed to satisfy food demand, they are also considerably affected by the significant increase in fertilizers under the optimized scenarios. By raising nutrients up to 270 kgN/(ha ·yr), noticeable infeasibilities only persist in the Banikoara region, although this is due to the very limited area dedicated to this crop, around 0.2% of total cropland. Maize is another important crop both for its value and for cereal food production. Under any of the baseline cropland scenarios BLS, it covers around 16% of the total agricultural area. In general, current crop distribution allows to locally produce enough cereals, except in Kérou, which is not self-sufficient for all cereals diet requirements, mainly because 70% of its crop area is used for cotton.

#### 4.3.2 Irrigation

Irrigation is very limited in the region, effectively restricted to some crops—mainly vegetables or rice—and specific areas close to surface waters along main rivers. Under scenario BLS_2025_FI200, irrigation is applied assuming that each crop and region can potentially benefit from its use. In doing so, we aim at detecting for what crop (and where) it would be more efficient to invest in water distribution and use. However, a more detailed analysis would be required here to accurately assess the transferability of the identified irrigation strategies. Nevertheless, it is interesting to note that under this assumption, several crops—actually not irrigated—would benefit from more irrigation use. On the other hand, vegetables is the crop with the highest water consumption—see the dark blue sectors in Fig. 7—with an approximate 31% global share, peaking to 67% in some areas of Niger. Other crops contributing to water depletion are rice (around 24%) and maize, cotton and tubers (all within 9–10%).

#### 4.3.3 Land allocation

In order to analyze the influence of the redistribution of crops on food security and benefit, three different land allocation optimization scenarios were identified and analysed by applying the DSS based on the BLS_2025_FI200 setting for other input (agricultural input management and food demand, see Table 4). The solution of the optimization process would identify a new optimal strategy for land allocation that is highly dependent on the constraints in force. Therefore, in order to take into account the impact of diversified constraints, three configurations with substantial different assumptions were proposed and assessed, as summarized in Table 5.

**Table 5:** Land allocation redistribution scenarios.

Land Constraint ID	Description
Cotton0to100	Cotton area unrestricted. Other crops cannot reduce their area, but they can increase up to 100%
Rest0to100	Cotton area constant. Other crops area unrestricted
BLS2025_10_60	Each crop area can vary within certain limits given by MinArea = min{10%areaReg, 60%CropAreaActual}. MaxArea = 100% region

**Fig. 8:**
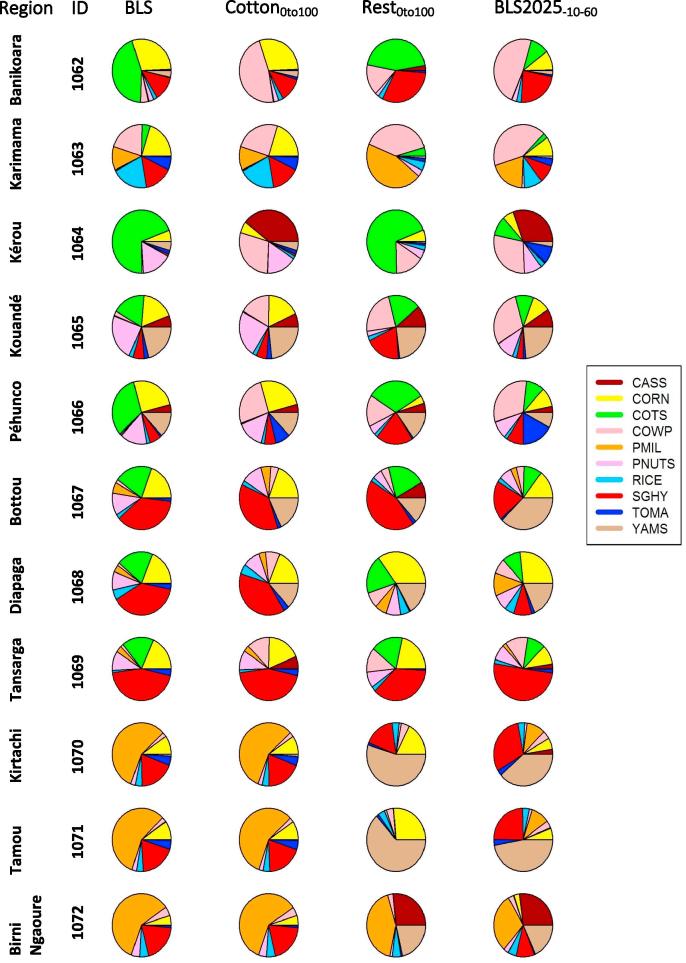
Optimal regional crop area redistribution for the scenarios considered.

Fig. 8 shows the optimal land allocation area for the three constraint scenarios proposed, together with the baseline scenario BLS2025. As we can observe, the area distribution varies significantly across scenarios. As expected, under the Cotton0to100 scenario, all the cropland used for cotton is replaced by food and highly productive and economically valued crops, like cowpeas (practically not present in the baseline scenario), tubers (cassava and yam) and, to a lesser extent, vegetables. In turn, areas initially used for cotton in Burkina Faso are now mostly sown with cowpea and tubers. Finally, no significant changes in the crop area distribution are observed in Niger, since cotton was not used under the baseline scenario.

As for the second scenario, Rest0to100, where cotton is assumed to be part of an established farming industry, an overall decrease in vegetables and dry cereals (maize, millet and sorghum), as well as a significant increase in tubers and cowpea, are observed. However, important differences arise when considering the regional level. To wit: (i) millet and sorghum, although globally decreasing, are more used in the Benin area; (ii) rice increases in the Burkina Faso region, but is reduced elsewhere; and (iii) yam is reduced in Benin (see e.g. region #1062 in Fig. 8), showing an opposite trend in other regions.

We finally consider the Rest10_60 scenario, where the possibility of reallocating cropland is more restricted, to account for local farmers’ reluctance to changes. The most evident feature is the gain in cropping area of tubers and cowpeas, although vegetables also show a significant increase, see regions #1064 and #1066 in Fig. 8. Rice is increased only in the Burkina Faso area (see region #1069 in Fig. 8), whereas, at the other extreme, sorghum is fostered in Benin and Niger, but reduced in Burkina Faso.

**Fig. 9:**
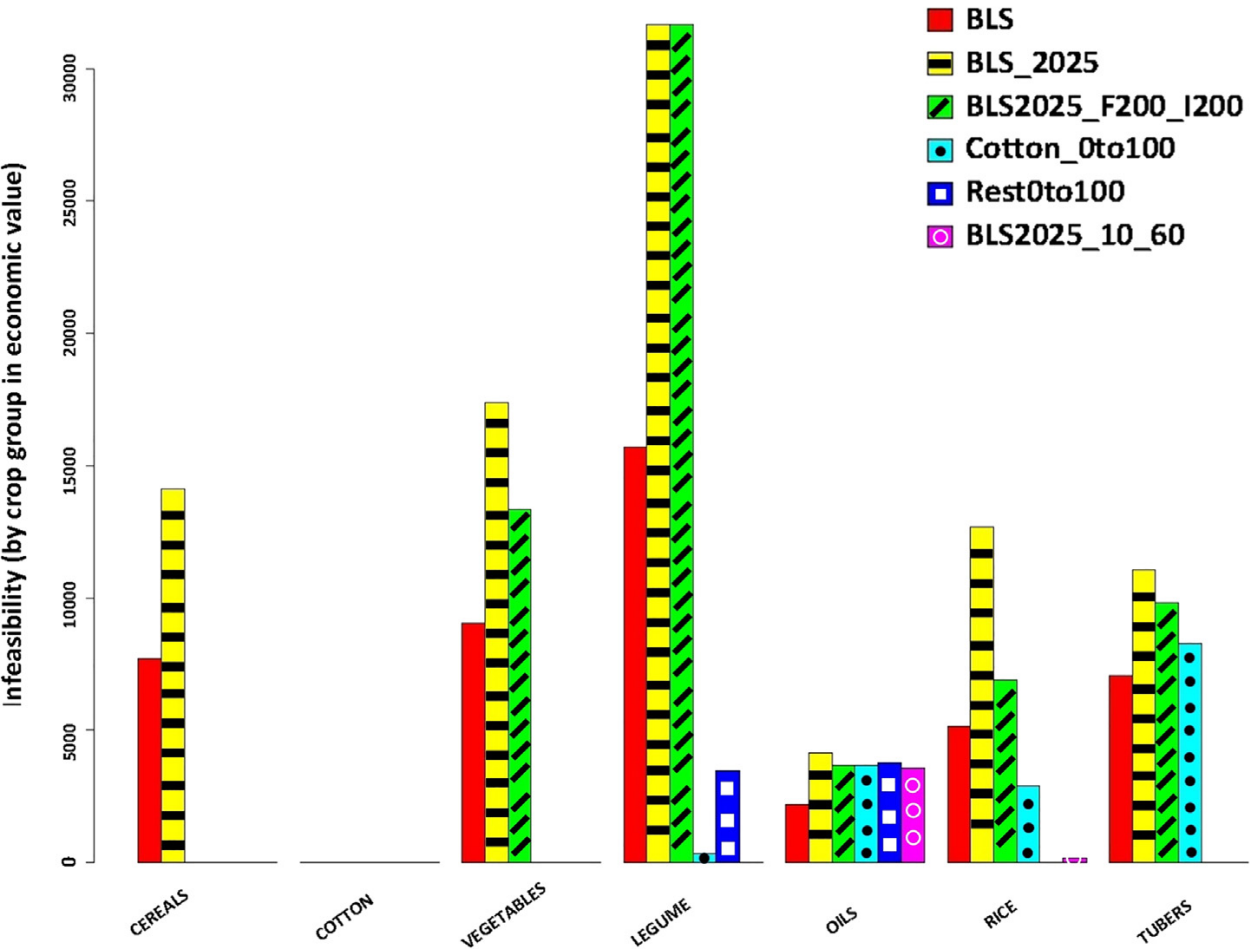
Comparison of total food security violation by crop group.

It is interesting to note that, while food security infeasibility is reduced in all the optimal scenarios considered, it is aggravated under the BLS2025 scenario due to the assumed population growth, see Fig. 9. On the other hand, irrigation and fertilization eradicate cereals infeasibilities and reduce significantly those in rice and vegetables. Optimal land allocation scenarios are highly effective, eliminating almost completely the issue of demand satisfaction in vegetable and leguminous. BLS2025_10_60 scenario is especially relevant, since all the infeasibilities disappear, except those of peanuts and, to a lesser extent, rice. As we can observe in Fig. 9, peanut deficit occurs in regions #1062 and #1063, where its growth is only increased when using irrigation but not fertilization. However, the main problems in those regions are the missing cropland (which is not enough to satisfy local food demands), and a very high initial infeasibility of vegetables.

**Fig. 10:**
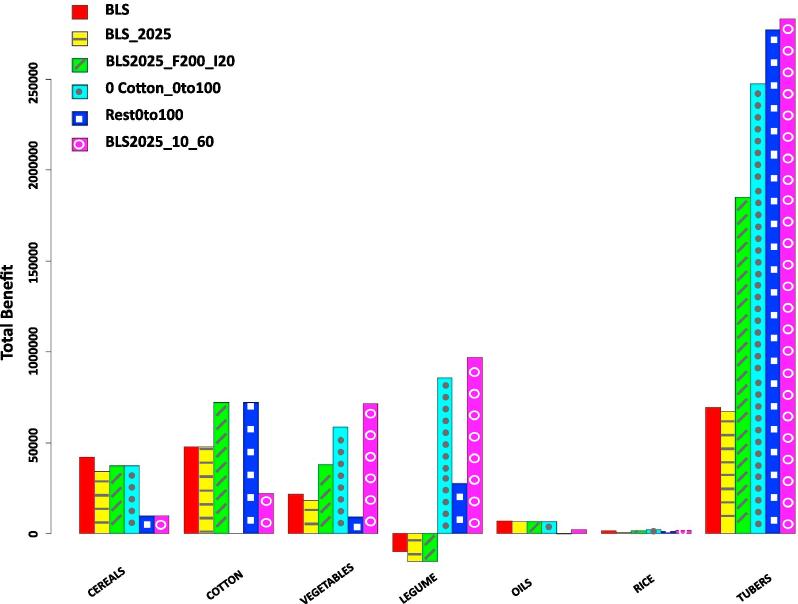
Comparison of total benefit by crop group.

Regarding the incomes obtained from each crop, tubers yield the maximum profit in all the optimized scenarios, equaling the aggregated benefit of the other crops, see Fig. 10. As we can observe in Fig. 8, areas dedicated to tubers and the corresponding applied fertilizer are significantly increased in many regions (up to 60% of the total area), something that would probably imply an oversupply of products and a drop-off in prices. Finally, note that some proposed solutions might not be realistic as, for instance, the Cotton0to100 food security scenario which, by completely replacing cotton, would theoretically have allowed to reduce food scarcity.

### 4.4 Sensitivity analysis

The selection of modeling parameters plays a crucial role in the analysis of results. Such parameters are usually subject to great uncertainty, mainly due to market fluctuations and extreme weather events. Sensitivity analysis has been adopted as a paradigm to evaluate parameters uncertainty and its influence (Li et al., 2015). Specifically, we have performed sensitivity analysis for the scenario providing best results in terms of lower infeasibilities and greater benefits, BLS2025_10_60. The following parameters were incorporated in the analysis, with variations of ±25% with respect to their base level: population variation, crop market price (in particular for yam, the most profitable crop), security factor, area constraints for land allocation, and fertilizer, irrigation water and total agricultural area availabilities.

**Fig. 11:**
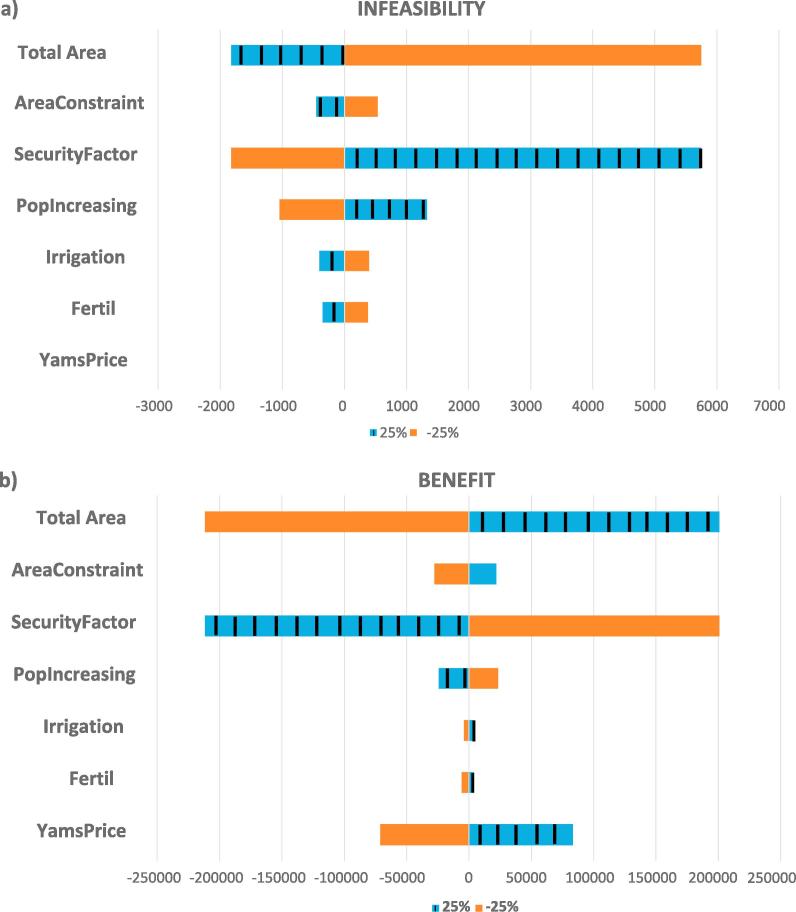
Sensitivity analysis of model parameters versus demand infeasibility (a) and versus the total benefit (b).

As shown in Fig. 11, the security factor and the total agricultural area have the largest impact on both food demand infeasibility and total benefit. Yam market price has also a great influence, since it is the key factor for gross income but, as expected, it does not affect food infeasibility.

## 5 Conclusion

In this paper, we have described the DSS module, part of the E-water tool. Specific attention has been devoted to the models and dataset underlying the DSS as well as to the results obtained from its application to the Mékrou river basin case study. The system was developed and applied to assess the Water Energy Food Ecosystem nexus, providing optimal management solutions at river basin level, in a context of food insecurity and increasing competition with other competing sectors. Specifically, we focused on the identification of optimal agricultural strategies for nutrient fertilizer and irrigation management, as well as on the optimal handling of modified cropland allocation and landuse.

Aiming to provide timely assessments, a simplified linear regression agricultural approach, based on the EPIC biophysical process, was developed and integrated into the optimization tool. A linear programing model and a multiobjective genetic algorithm were also designed to perform tradeoff analysis among multiple and conflicting objectives, related to agricultural productivity, food security and natural water resources exploitation. All these components were embedded in a friendly user interface allowing stakeholders, decision makers and local expert technicians to easily build and customize new management and application scenarios.

The developed E-Water software is open source, since it is intended to be applied in developing countries, see https://aquaknow.jrc.ec.europa.eu/. The tool can help analyze agronomic planning by finding efficient patterns of fertilization, irrigation and land allocation, subject to various environmental and practical considerations, such as water exploitation, climate change, fertilizer available, population demand or limited available agricultural areas. The system is designed to seek solutions primarily prioritizing the minimization of food security, and aiming at maximizing the production surplus as a secondary objective. The tool also includes a sensitivity analysis module to assess robustness of results.

Our model provides quantitative analyses of management decisions, as given by irrigation and fertilization patterns for each crop and region. For instance, it is estimated that the foreseen population growth—and the implied increase in food demand—by 2025 would trigger food infeasibility by 95%. However, that could be significantly attenuated by effectively applying fertilizer and irrigation (up to 30% for some regions and crops), while increasing, at the same time, benefits by 105%. The identification of optimal agricultural management strategies resulting from the increased use of fertilizers and irrigation has highlighted the local capacity to produce more food items. Nevertheless, important deficits still remain, limiting the production capacity to completely satisfy local food demand. Aiming to gain more insight, we explored three alternative scenarios—focusing on optimal cropland allocation—to assess whether different uses of existing cropland could increase food self-sufficiency. Under the assumptions that cash crops can be entirely abandoned, and that other food crops can be easily interchanged, the analysis resulted in: (i) a nearly total eradication of local incapacity to produce enough crops to satisfy food demand; and (ii) a noticeable increase in profit, between 18% and 38%.

Optimal cropland allocation scenarios were found to be very dependent on the restrictions considered, thus requiring the direct involvement of local stakeholders to effectively define them (Markantonis et al., 2017b). In this regard, we acknowledged that even when fixing the area dedicated to cash crop cotton (thus limiting the possibility to increase cropland for food crops), significant improvements were still achieved regarding food self-sufficiency. Indeed, the redistribution of current food cropland allowed an additional 15% reduction in the food infeasibility indicator, compared with the optimal scenario, where only fertilizer and irrigation were optimized, while land allocation was kept constant.

We performed an illustrative sensitivity analysis for the BLS2025_10_60 scenario, showing that food security was especially affected by several factors, as the cropland area available, the population growth rate the change in diet, and crop selling prices. This highlights the need to characterize the parametrization of the scenarios and the definition of constraints in a precise way.

We end up discussing the issue of availability of data. At the time of completing the current tool design, only crop incomes were included in the evaluation of the total benefit. In turn, there was no reliable information about costs associated with agricultural practices, and, hence, they were disregarded. However, should these data eventually become available, they could be easily integrated into the DSS. Although they would not affect significantly the assessment of food security, they could, however, substantially change the identification of optimal strategies in relation with the added benefit resulting from the surplus production.

## Footnotes

1 Note the use of the following abbreviations: tropical fruit-BANA, cassava-CASS, maize-CORN, cotton-COTS, cowpea-COWP, millet-PMIL, peanuts-PNUT, potatoes-POTA, rice-RICE, sorghum-SGHY, vegetables-TOMA and yams-YAMS
